# Honey bee venom re-challenge during specific immunotherapy: prolonged cardio-pulmonary resuscitation allowed survival in a case of near fatal anaphylaxis

**DOI:** 10.1186/s13223-022-00687-x

**Published:** 2022-06-02

**Authors:** Sara Micaletto, Kurt Ruetzler, Martin Bruesch, Peter Schmid-Grendelmeier

**Affiliations:** 1 Allergy Unit, Dept. of Dermatology, University Hospital and University of Zürich, Zurich, Switzerland; 2 Institute for Anesthesiology, University Hospital and University of Zürich, Zurich, Switzerland; 3 Cleveland Clinic, Anesthesiology Institute, Departments of Outcomes Research and General Anesthesia, Cleveland, OH, USA

**Keywords:** Specific immunotherapy, Honey bee, Re-challenge, Anaphylaxis, Cardio-pulmonal resuscitation

## Abstract

**Background:**

Specific immunotherapy for patients with honey bee hypersensitivity is commonly applied. Re-challenge with venom is performed to prove protection in individual cases.

**Case presenation:**

We report a case of near fatal anaphylaxis with asystole for 24 min in a 35-years-old patient with mastocytosis after honey bee sting challenge, despite 5-years of specific immunotherapy. Successful cardio-pulmonary resuscitation was applied for 32 min.

**Conclusion:**

This intervention demonstrates, that in anaphylaxis with cardio-vascular arrest, prolonged cardio-pulmonary resuscitation for up to 40 min may be appropriate to overcome the half-life of massively released histamine. Failure of specific immunotherapy was possibly due to sensitization to the allergen *Api m10*, potentially underrepresented in commercial honey bee venom extracts. Molecular analyses may provide additional clues to the potentially unsuccessful outcome of venom specific immunotherapy, especially in high-risk patients such as mastocytosis.

## Background

Hypersensitivity to insect venom is a common cause for severe to life-threatening anaphylaxis and affects up to 8.9% of the population [1]. Honey bees (*Apis mellifera*) or yellow jackets (Vespula germanica and Vespula vulgaris) lead to systemic anaphylactic sting reactions in the majority of cases [2]. Mastocytosis is a risk factor for severe anaphylactic reactions in patients allergic to hymenoptera [3]. Venom immunotherapy (VIT) is highly effective in preventing further systemic anaphylactic sting reactions, however, 2.4%–20.4% of those allergic to bee venom or yellow jacket venom were not protected when VIT was performed for 7–38 months [4]. In individual cases, re-challenge is of importance to prove protection [5].

## Case presentation

We report on a 35-year-old patient, who experienced a severe anaphylactic reaction after a honey bee sting in 2007, including hypotension and loss of consciousness (Grade IV reaction). Sensitization to bee venom (BV) was confirmed by detection of elevated, allergen-specific IgE in serum (i1, 24 kU/l) and positive intradermal skin test (0.01 µg/l), while no specific IgE against wasp (i3) were detectable. Thus, venom specific immunotherapy (VIT) with bee venom extract was initiated in June 2008 and performed continuously according to international guidelines with 100000 SQE every 4 weeks, and was well tolerated by the patient [4, 5].

In January 2013 and after almost 5 years of immunotherapy, a clinical and laboratory follow-up was performed. Specific IgE antibodies for bee venom (i1) decreased from 24 to 6 kU/l, while specific IgG increased as expected after 5 years of VIT (Table 1). Unexpectedly, the serum tryptase level, that was within normal range in 2008 (3.3 µg/l), was now elevated (21 µg/l) [6, 7]. Serum tryptase measurement was performed 14 days after last VIT, and no prior stinging incident or allergic reaction. Further investigations revealed minor teleangiectesias on the trunk and swelling opon mechanical pressure (i.e. positive Darier’s sign). In addition, a skin biopsy was performed and revealed increased numbers of mast cells, compatible with cutaneous mastocytosis [7]. Apart from sporadic dizziness, no symptoms further indicating systemic mastocytosis were reported by the patient and c-Kit mutation was not detectable from the tissue biopsy. However, increased abnormal mast cells > 25% and expression of aberrant CD25 cells was detectable in a bone marrow aspirate, two minor criteria of systemic mastocytosis [8].

**Table 1:** Patient serum analysis on molecular sensitization patterns

	rApi m1	rApi m2	rApi m3	rApi m4	rApi m10
Patient serum analysis (time of sting provocation)					
IgE kU/l	89.2	> 100	1.85	9.94	10.5
IgG4 ug/l	> 50,000	7285	272	> 50,000	241

	rApi m1	rApi m2	rApi m3	rApi m4	rApi m10
Comparison data: serum of a protected patient					
IgE kU/l	1.6	15.9	0.22	2.3	2.42
IgG4 ug/l	> 50,000	15,190	19,320	> 50,000	1800

	rApi m1	rApi m2	rApi m3	rApi m4	rApi m5	rApi m10	Date**
Patient serum analysis (time of sting provocation; 1 month later; after 6 months of VIT)							
IgE kU/l	89.2	> 100	1.85	9.94	9.9	10.5	T + 3 days
IgG4 ug/l	> 50,000	7285	272	> 50,000	1890	241	T + 3 days
IgG4 ug/l	> 50,000	7692	339	> 50,000	2470	533	T + 28 days
IgG4 ug/l	> 50,000	10,640	1716	> 50,000	3834	< 200	T + 7 months *
IgG4 ug/l						< 200	T + 4 years°

*6 months after reaching triple VIP maintenance dose (100000 SQE plus 300 μg HBV concomitant with Omalizumab)

**T stands for day of sting challenge

°in between well tolerated 3 years after sting challenge, under VIT mot Omalizumab and 200 ug HBV

The patient was working as a gardener and ultimately wanted to know his level of protection under ongoing VIT, given his very high risk of re-stings, and also knowing that patients with a tolerated sting challenge have a better quality of life [9]. It is known, that patients with mastocytosis are at higher risk for severe or even fatal anaphylaxis, after stopping or even during VIT [10].

Due to his current immunological profile, with decreased specific IgE and increased IgG4 levels against BV since starting VIT, we performed a bee venom sting challenge, according to the patients wish with the clear consensus that we will continue VIT irrespective of the sting challenge outcome. The sting challenge was performed under all necessary precaution measures and according to international guidelines for safety measurements, 10 days after his last injection of VIT maintenance dose. Specifically, the patient was monitored with ECG, non-invasive blood pressure and pulse oximetry and intravenous access was established. The sting was performed on the patient’s forearm and the stinger was left for 1 min in situ before being removed.

Four minutes after the sting, the patient developed generalized flushing, nausea, followed by cramps and emesis. Despite rapid administration of two doses of 0.3 mg intramuscular epinephrine as well as 250 mg methylprednisolone and 2 mg clemastine (both intravenous), the condition of the patient deteriorated continuously. Within minutes severe anaphylaxis developed, including dyspnea, followed by tachycardia and cardiac arrest. Cardio-pulmonary resuscitation (CPR) according to current ERC guidelines was immediately started. Within two minutes, the hospital resuscitation team was present and performed advanced CPR treatment. Due to SpO2 levels around 60% and clinical signs of central hypoxia, assisted ventilation was initiated. Non-invasive, peripheral blood pressure could not be measured and central carotid pulse was barely palpable. Electrocardiographic analysis revealed 2^nd^ degree AV-block rapidly followed by ventricular fibrillation. Accordingly, defibrillation was performed (biphasic, 200 J) and CPR was continued, including securing the patient’s airways by tracheal intubation [11, 12]. Rhythm analysis after defibrillation showed pulseless electric activity (PEA) for further 4 min followed by asystolia for cumulative 24 min, during which CPR was continued. Finally, ventricular fibrillation recurred leading to immediate defibrillation. After 32 min of CPR, and a total of 10 mg epinephrine intravenously and 2000 ml isotonic fluid, return of spontaneous circulation (ROSC) occurred. Amiodarone, fentanyl, rocuronium, propofol and midazolame were intravenously administrated and the patient was hemodynamically stabilized. The patient was then transferred to the emergency department, where a central venous and arterial catheter were inserted and a cumulative 1500 ml isotonic fluid administrated (results of initial and before discharge arterial analyzes are summarized in Table 2.). The patient was finally transferred to intensive care unit, where therapeutic hypothermia for 24 h was initiated, amongst other treatment modalities.

**Table 2:** Pathophysiologic changes during and after CPR

	Initial analyze (time: 12:26)	Admission to ICU (time: 13:13)
pH	7.058	7.197
pCO_2_ (kPa)	5.93	5.51
pO_2_ (kPa)	26.1	28.4
Hkt (%)	0.543*	0.548*
Hb (mmol/l)	17.7	17.9
Lactat(mg/dl)	11.2	6.2
Glucose (mmol/l)	18	15.1
HCO_3_ (mmol/l)	11.9	15.5
Base excess	−19.5	−12.4
Total fluid administration	2000 ml	3500 ml

^*^Demonstration of vasoplegia, as a direct consequence of anaphylactic shock

The patient recovered slowly, initially showing transitory signs of post-traumatic stress disorder, including reduced fine motor skills and concentration difficulties. After several months of rehabilitation, cognitive functions and motor skills steadily improved. No permanent sequalae remained and the patient was able to return to his former employment.

After recovery from the anaphylactic incident and rehabilitation, and in addition of the ongoing VIT treatment (300 µg honey venom extract, q4w), s.c. Omalizumab treatment (300 mg, q4w) was initiated. During the currently ongoing combinatorial treatment, the patient has been stung several times by honey bees, without any signs of anaphylaxis. Interestingly, levels of IgG4 against *Api m10* remained low.

## Discussion and conclusion

Duration of CPR theoretically corresponds partly to the half-life of histamine to overcome circulation-relevant effects [13, 14]. Maximum plasma histamine levels are found 20–30 min after allergen challenge and were shown to correlate with the severity of anaphylaxis [15, 16]. In our case, tryptase was measured two hours after sting challenge and revealed excessively increased plasma levels to around 1300 μg/l, confirming an extreme degranulation of mast cells [3]. This fulfills all criteria of mast cell activation (MCA), requiring a 20% increase from physiological baseline tryptase levels [3]. Four days later, plasma tryptase levels decreased to 10.4 µg/l, and later stabilized at 16 µg/l.

Hymenoptera venom typically contains a mixture of 3–4 major proteins as well as pharmacologically active peptides and other small molecules. There are common proteins but also significant differences amongst the various Hymenoptera species [17]. The possibility to measure specific IgE on a molecular pattern has substantially contributed to a better understanding of allergologic mechanisms in recent years [18]. A detailed analysis of molecular sensitization patterns was performed and showed a clinically-relevant sensitization, not only to major honey bee allergens *Api m1* and *Api m2*, but also to *Api m3* and an even higher level to *Api m10*, amongst others (see Table 1). Previous studies demonstrated that commercial extracts seem to contain only very limited amounts of *Api m10*, in contrary to natural honey bee venom [19]. In the present case, retrospective serological profiling revealed, that after 5 years of VIT, allergen-specific IgG to *Api m1* and *Api m2* increased, while allergen-specific IgG to *Api m10* did not, reflecting possible insufficient immune response to *Api m10*. Arguing, that this may be partly due to the lack of sufficient *Api m10* allergen in 150 µg non-aqueous bee-venom extract, VIT maintenance dose was increased after the near-fatal sting challenge to 300 µg bee venom by adding 200 µg honey-bee venom extract in aqueous solution, potentially containing higher amounts of *Api m10*. In addition, the patient received additional preventive treatment, including H1- and H2-blockers, ketotifen and omalizumab. After 6 months of VIT with a total dose of 300 ug honey bee venom every 4 weeks (and Omalizumab) the patient showed an increase of IgG4 to *Api m3* and *Api m5.* However, no increase in IgG4 levels to *Api m10* was detectable, while in comparison, a patient suffering from mastocytosis and sufficiently protected by VIT (confirmed by a sting challenge), showed relevant titers of *Api m10* specific IgG4 (see Table 2). Thus, mismatch between sensitization to allergens such as *Api m10* might explain severe anaphylaxis occurring after a bee sting in mastocytosis patients despite performed VIT. As recently suggested for food allergens [20, 21], and seen in this case, lack of allergens may only be partly compensated by dose escalation if extracts are not truly “spiked” with the relevant lacking allergens. In the obvious absence of protective IgG4 antibodies against *Api m10,* the question is, whether Omalizumab is protecting the patient by modulating the IgE-dependent immune response or whether *Api m10* did not play an important role in the near fatal reaction after the sting challenge—as seen in other studies where patients with exclusive and absent IgG4 response to *Api m10* also tolerated re-stings. Data from the use of Omalizumab as premedication for honey bee VIT with severe side effects underscore its potential therapeutic effect [22]. Additionally, Omalizumab is reported to be protective in patients with mastocytosis and anaphylaxis in bee keepers not protected by conventional VIT with HB [23, 24].

We conclude the following for the clinical application of VIT: the decision for re-challenge must be taken with extreme care and only should be considered only for a minority of patients, that have high-risk features regarding medical and/or life constellations. Re-challenges to hymenoptera venom should only be performed by highly specialized and well-trained staff with all measures in place to start immediate CPR in case of severe anaphylaxis, and all the resources to quickly escalate treatment to advanced CPR and ICU transfer. Importantly, in case of hymenoptera-induced anaphylaxis with cardio-vascular arrest, prolonged CPR for up to 40 min may be appropriate to overcome the half-life of massively released histamine and other cardio-vascular active mediators. In patients with bee venom allergy, extensive molecular analyses may provide additional insight to potential unsuccessful outcome of VIT, due to allergen mismatch between bee venom and extract [25]. Finally, Omalizumab should be considered as an additional line of treatment in high-risk patients during honey bee VIT.

## Data Availability

Not applicable.

## References

[1] Bilò MB, Bonifazi F (2009). The natural history and epidemiology of insect venom allergy: clinical implications. Clin Exp Allergy.

[2] Bilò MB (2011). Anaphylaxis caused by Hymenoptera stings: from epidemiology to treatment. Allergy.

[3] Schwartz LB, Metcalfe DD, Miller JS (1987). Tryptase levels as an indicator of mast-cell activation in systemic anaphylaxis and mastocytosis. N Engl J Med.

[4] Ruëff F, Wenderoth A, Przybilla B (2001). Patients still reacting to a sting challenge while receiving conventional Hymenoptera venom immunotherapy are protected by increased venom doses. J Allergy Clin Immunol.

[5] Pesek RD, Lockey RF (2014). Treatment of Hymenoptera venom allergy: an update. Curr Opin Allergy Clin Immunol..

[6] van Doormaal JJ, van der Veer E, van Voorst Vader PC (2012). Tryptase and histamine metabolites as diagnostic indicators of indolent systemic mastocytosis without skin lesions. Allergy.

[7] Pardanani A (2013). Systemic mastocytosis in adults: 2013 update on diagnosis, risk stratification, and management. Am J Hematol.

[8] Valent P, Akin C, Arock M (2012). Definitions, criteria and global classification of mast cell disorders with special reference to mast cell activation syndromes: a consensus proposal. Int Arch Allergy Immunol.

[9] Fischer J, Teufel M, Feidt A (2013). Tolerated wasp sting challenge improves health-related quality of life in patients allergic to wasp venom. J Allergy Clin Immunol.

[10] Oude Elberink JN, de Monchy JG, Kors JW (1997). Fatal anaphylaxis after a yellow jacket sting, despite venom immunotherapy, in two patients with mastocytosis. J Allergy Clin Immunol.

[11] Travers AH, Rea TD, Bobrow BJ, Edelson DP, Berg RA, Sayre MR (2010). Part 4: CPR overview: 2010 American heart association guidelines for cardiopulmonary resuscitation and emergency cardiovascular care. Circulation.

[12] Deakin CD, Nolan JP, Soar J, Sunde K, Koster RW, Smith GB (2010). European resuscitation council guidelines for resuscitation 2010 Section 4. Adult advanced life support. Resuscitation.

[13] Simons FE, Frew AJ, Ansotegui IJ (2007). Risk assessment in anaphylaxis: current and future approaches. J Allergy Clin Immunol.

[14] Schwartz LB, Atkins PC, Bradford TR, Fleekop P, Shalit M, Zweiman B (1987). Release of tryptase together with histamine during the immediate cutaneous response to allergen. J Allergy Clin Immunol.

[15] Triggiani M, Patella V, Staiano RI (2008). Allergy and the cardiovascular system. Clin Exp Immunol.

[16] Vadas P, Gold M, Perelman B (2008). Platelet-activating factor, PAF acetylhydrolase, and severe anaphylaxis. N Engl J Med.

[17] Pesek RD, Lockey RF (2013). Management of insect sting hypersensitivity: an update. Allergy Asthma Immunol Res.

[18] Canonica GW, Ansotegui IJ, Pawankar R (2013). A WAO–ARIA–GA^2^LEN consensus document on molecular-based allergy diagnostics. World Allergy Organ J.

[19] Blank S, Seismann H, Michel Y (2011). Api m 10, a genuine A. mellifera venom allergen, is clinically relevant but underrepresented in therapeutic extracts. Allergy.

[20] Zuidmeer-Jongejan L, Fernández-Rivas M, Winter MG (2014). Oil body-associated hazelnut allergens including oleosins are underrepresented in diagnostic extracts but associated with severe symptoms. Clin Transl Allergy.

[21] Kattan JD, Sicherer SH, Sampson HA (2014). Clinical reactivity to hazelnut may be better identified by component testing than traditional testing methods. J Allergy Clin Immunol Pract.

[22] Gülsen A, Ruëff F, Jappe U (2021). Omalizumab ensures compatibility to bee venom immunotherapy (VIT) after VIT-induced anaphylaxis in a patient with systemic mastocytosis. Allergol Select.

[23] Kontou-Fili K, Filis CI (2009). Prolonged high-dose omalizumab is required to control reactions to venom immunotherapy in mastocytosis. Allergy.

[24] Sokol KC, Ghazi A, Kelly BC, Grant JA (2014). Omalizumab as a desensitizing agent and treatment in mastocytosis: a review of the literature and case report. J Allergy Clin Immunol Pract.

[25] Frick M, Müller S, Bantleon F (2015). rApi m 3 and rApi m 10 improve detection of honey bee sensitization in Hymenoptera venom-allergic patients with double sensitization to honey bee and yellow jacket venom. Allergy.

